# Toward Clinically Actionable Explainable AI in Pulmonary Arterial Hypertension: Endpoints, Calibration, and External Validation. Comment on Ledziński et al. Personalized Medicine in Pulmonary Arterial Hypertension: Utilizing Artificial Intelligence for Death Prevention. *J. Clin. Med.* 2025, *14*, 8325

**DOI:** 10.3390/jcm15051719

**Published:** 2026-02-25

**Authors:** Gianluca Pagnoni, Aurora Vicenzi, Francesca Coppi

**Affiliations:** 1 Cardiology Unit of Emergency Department, Guglielmo da Saliceto Hospital, 29121 Piacenza, Italy; 2 National Institute for Cardiovascular Research (INRC), Via Irnerio 48, 40126 Bologna, Italy; 3 Department of Medical and Surgical Sciences for Children and Adults, University of Modena and Reggio Emilia, Via del Pozzo 71, 41124 Modena, Italy

We read with great interest the recent contribution proposing a machine-learning model (XGBoost), developed using registry data, to estimate mortality risk in adult patients with pulmonary arterial hypertension (PAH) and incorporating an SHAP-based interpretability strategy to clarify, both globally and at the individual level, the determinants of the prediction. This approach is particularly valuable because it supports a clinically credible use of artificial intelligence: not a “black box” producing a score, but rather a tool capable of substantiating its output and thus engaging with medical reasoning and clinical communication [1].

Nevertheless, several methodological and translational aspects deserve consideration, as they may influence real-world implementation. First, the selected endpoint (“death by the next follow-up visit”), in the presence of non-uniform follow-up intervals, may introduce a component driven by the healthcare organization in addition to intrinsic prognosis; in this regard, adopting predefined time horizons (e.g., 6–12 months) or a time-to-event framework could improve comparability and clinical usefulness [1]. Supporting this view, prognostic analyses in populations with pulmonary hypertension associated with chronic respiratory diseases are commonly conducted using survival models, identifying independent predictors related both to hemodynamic severity and functional measures, thereby providing a natural methodological reference for temporally standardized endpoints [2]. Second, while prioritizing high sensitivity is understandable in a setting where the clinical cost of false negatives is substantial, a non-negligible proportion of false positives (with consequent reduced precision) makes it essential to define the intended use case and downstream “action pathway” explicitly: which interventions should follow a “high-risk” classification and what balance is expected between clinical benefit and healthcare burden. A similar message arises in PAH screening among high-risk populations, where algorithms with high sensitivity and negative predictive value can reduce missed diagnoses but require structured diagnostic-decision pathways to manage lower specificity and the risk of over-testing appropriately [3]. Finally, for practical applicability, beyond discrimination metrics, it would be desirable to report calibration comprehensively and to discuss the decision threshold transparently, since well-calibrated probabilities and clinically justified thresholds are pivotal for turning predictions into decisions [1].

From a fully “personalized” perspective, it also appears important to consider phenotypic determinants that may affect presentation, diagnostic accuracy, and treatment response. In cardiovascular medicine, sex-specific differences in diastolic dysfunction and heart failure with preserved ejection fraction (HFpEF) have been described, with practical implications for diagnostic criteria, imaging interpretation, and therapeutic strategies—a dedicated reflection on how such dimensions might be integrated into predictive modeling could strengthen the clinical translation of the results [4]. Complementarily, in systemic conditions at high risk of cardiopulmonary involvement, potentially modifiable biological factors are associated with relevant echocardiographic parameters: vitamin D insufficiency, for instance, has been linked to a less favorable cardiovascular risk profile and to echocardiographic indices consistent with greater cardiopulmonary impairment, suggesting that prognostic stratification may benefit from an integrated assessment that also includes modifiable determinants [5]. Moreover, in different scenarios sharing the central role of right ventricular function and pulmonary afterload, non-invasive hemodynamic indices may provide prognostic information; in acute pulmonary embolism, an echocardiography-derived pulmonary artery pulsatility index (PAPI) has been associated with short-term adverse outcomes, supporting the interest of integrating targeted echocardiographic variables into risk models when the goal is to anticipate clinical deterioration [6].

In conclusion, the study provides a relevant contribution to the discussion on integrating real-world registries and explainable artificial intelligence in PAH. To consolidate its impact, we consider the following priorities: temporally standardized endpoints or time-to-event approaches, comprehensive assessment of calibration and decision thresholds, external validation in independent cohorts, and a more structured discussion of phenotypic determinants, including sex-specific features and potentially modifiable factors, that may influence interpretation and clinical use of predictions.
